# Harnessing Bio-Immobilized ZnO/CNT/Chitosan Ternary Composite Fabric for Enhanced Photodegradation of a Commercial Reactive Dye

**DOI:** 10.3390/molecules28186461

**Published:** 2023-09-06

**Authors:** Usama Bin Humayoun, Fazal Mehmood, Yasir Hassan, Aamir Rasheed, Ghulam Dastgeer, Asad Anwar, Nasir Sarwar, Daeho Yoon

**Affiliations:** 1 Department of Textile Engineering, University of Engineering & Technology, Lahore (Faisalabad Campus), Faisalabad 38000, Pakistanasad.anwar@uet.edu.pk (A.A.); 2 Department of Materials Science and Engineering, Chungnam National University, Daejeon 34134, Republic of Korea; 3 School of Materials Science and Engineering, Anhui University, Hefei 230022, China; 4 Department of Physics and Astronomy, Sejong University, Seoul 05006, Republic of Korea; gdastgeer@sejong.ac.kr; 5 School of Advanced Materials Science and Engineering, Sungkyunkwan University, Suwon 16419, Republic of Korea; 6 SKKU Advanced Institute of Nanotechnology (SAINT), Sungkyunkwan University, Suwon 16419, Republic of Korea

**Keywords:** immobilized photocatalysts, photocatalysis, sustainable wastewater treatment, bilateral esterification, ZnO

## Abstract

Growing demand for sustainable wastewater treatment drives interest in advanced photocatalytic materials. Immobilized photocatalysts hold potential for addressing industrial wastewater organic pollutants, offering substantial surface area, agglomeration prevention, and easy removal. In this study, we successfully immobilized ZnO and carbon nanotubes onto a textile substrate through bilateral esterification and explored their effectiveness as a potent photocatalyst for degrading of commercial textile colorant reactive blue 4 (RB-4) colorant. Findings demonstrated significant improvements in photocatalytic performance upon integrating ZnO and CNTs into the fabric, coupled with chitosan immobilization. The immobilization process of ZnO and CNTs onto the substrate was elucidated through a proposed reaction mechanism, while the appearance of carbonyl peaks at 1719.2 cm^−1^ in the composite fabric further confirmed bilateral esterification. The as-developed immobilized nano-catalyst exhibited remarkable photocatalytic efficiency with an impressive 93.54% color degradation of RB-4. This innovative approach underscores the immense potential of the ternary immobilized (ZnO/fCNT/chitosan) composite fabric for efficient photocatalytic degradation in textile coloration processes. Exploring the early-stage development of immobilized photocatalysts contributes to safer and more eco-friendly practices, addressing pressing environmental challenges effectively.

## 1. Introduction

Industrialization and technological advancement outfield the human progress in every field of life with huge contribution in economy. Unfortunately, this tremendous progress is achieved at a cost of excessive damage to natural habitat, water contamination and environmental pollution. The textile industry is ranked the second biggest global economic activity with a total contribution of 1.8 trillion US dollars, but it also counts as the world’s second most pollutant industry [1,2]. Environmental pollution caused by the textile and leather industry is multi-folded with a worse impact on the ecosystem worldwide. On the one hand, it consumes huge amounts of water, which not only causes remarkable reduction in drinkable water amounts, but also contaminates the freshwater with its toxic drain. According to the Natural Resources Defense Council (NRDC), approximately 25% of the chemicals manufactured globally are applied in the textile industry [3]. The cleaning of and caring for cloths during the course of utilization also contribute to the ecological burden. Conventional washing requires 170–190 L of water against the capacity of per cubic ft (for consumer cloths) which carries washing pollutants including colorants [4]. Textile dyes and colorant subjected to their high water solubility and complex organic structure are difficult to remove or degrade [5]. Considering the current water scarcity and burgeoning human population, clean water and its reutilization are the ultimate need of the time. In recent years, a series of new technologies and methods have been developed for removal of organic dyes in traditional printing and dyeing wastewater, mainly including physical separation, biodegradation and chemical treatment. Among them, physical methods can separate or transfer pollutants through membrane separation technology but may bring secondary pollution. The development of biodegradation technology is limited because of its narrow application range and high cost. In chemical treatment, ozone oxidation, photocatalytic oxidation and electrochemical oxidation can be used to decompose organic dyes. Among various technological advancement in the field of wastewater treatment, nanomaterials have been gaining importance in connection to photodegradation of organic dyes. In this regard, the transition metal oxide (TMOs) nanoparticles, especially ZnO, TiO_2_, CaO, WO_3_, ZnS, SiO_2_, and SnO_2_, have been widely reported in the literature and found effective in decolorization of dyes [6,7,8]. On interaction with the wastewater, the colorant first absorbs on the material surface due to electrostatic interaction [9]. Upon irradiation with UV light, visible or sun light, metal oxides produce electron–hole pairs through the excitation of an electron from the valance band to the conduction band. This generated pair produces free radical species on reacting with moisture and air in the environment, eventually destroying the organic contaminations and colorant through redox reactions [10]. The mechanism of photodegradation on TMOs is well documented in the literature and has extensively been studied against various industrial and biological applications [11,12]. However, quick agglomeration of the homogenous TMOs nanoparticles, rapid recombination of generated electron–hole pair and prolonged irradiation time limit their practical utilization in wastewater treatment [5,13]. Taking the advantage of easy excitation subjected to low band gap, the hetero-structured TMOs are widely explored these days for water purification.

The advanced oxidation mechanism arises by unique multi-material heterostructures with delayed electron–hole pair combination and large surface area provide the competitive edge over homogenous phase with effective and rapid degradation. Among TMOs, TiO_2_ and ZnO heterostructure are excessively explored for photodegradation. Heterostructures involving various transition metal oxides (TMOs) with 2D carbon materials have also been investigated in the literature to showcase improved effectiveness compared to their individual metal oxide counterparts [9,14]. Aamir et al. employed ZnO/Mexen heterojunctions for effective photodegradation of Methylene blue (MB) [15]. Weibing et al. explained the quick electron transfer phenomenon in ZnO/Carbide (Mexen) photocatalysts [16]. Although the reported literature presents effective photodegradation of organic colorants, their synthesis involves complex and lengthy synthesis process [17]. Further, the synthesis of Mexen includes utilization of toxic HF acid, whereas the stability of carbide materials in aqueous medium is also questionable [18]. Likewise, 2D carbon materials, heterojunction of TMOs with carbon nanotubes (CNT), were also explored to tune up the optical band gap of the nano catalysts with extended performance and stability. Multiwalled carbon nanotubes have widely been used with ZnO and TiO_2_ [19,20]. The ability of CNT to continuously accept the electron from TMO inhibit the recombination of electron–hole pairs, hence presenting CNT/TMO as better photocatalysts compared to respective pure materials. However, functionalization on CNT is usually performed with toxic acids [21]. Bimetallic TMO structures (TiO_2_,/ZnO, SiO_2_/ZnO, SnO_2_/ZnO or TiO_2_ etc.) [6], 3D metal ion doping (Ti, Sc, V, Sc, Ni, V, Mn, Cr, Co, Fe, Co etc.) [22,23] and dye sensitized TMO have also been explored to upsurge the photodegrading performance [24]. Yi et al. manufactured the ZnO-NFKs and GO composite using a precipitation technique. Due to interface coupling, the electrons are transferred from the ZnO valance band to the GO band, resulting in a 97.6% photodegradation. Ong et al. formed a ZnO network with catalytically active faces after preparation of a composite with Ag and Au. N. Kumaresan et al. developed a (ZnO/CuO)@rGO heterostructure by solid-state synthesis. The composite showed efficient degradation of the RhB dye. Kanakkillam et al. fabricated a composite of cobalt oxide and ZnO NFKs that was exceptionally stable with rapid degradation performance. All the reported literature reciprocates the textile wastewater treatment with their prepared nanomaterials. It is unlikely that all the reported studies mostly utilize nonconventional colorants to evaluate the decolorization capacity of their prepared materials [5,25]. Further, their synthesis either involves utilization of toxic reagents or prolong process time [17] while the weak material (photocatalysts) to pollutant (colorant) interactions are the other challenges that the researchers are trying to overcome [26]. Moreover, after the completion of reaction time, the removal of nanomaterials is still very challenging and requires a complex filtration process while the unfiltered nanoparticles remaining in the solution act as an added source pollutant and contribute to heavy metal ion concentration [27]. In search of the stated issue, immobilized photocatalysts are now explored as a new class of photocatalysts [28]. The term immobilization is employed to photocatalysts anchored to a suitable substrate to restrict their release and agglomeration. The well-dispersed and firmly bonded nanomaterial suffers least physical deterioration and less aggregation as a consequence of the improved performance. Therefore, for now, the research on immobilized structures is at early stages [29] while most of the reported work is based on packing films and hydrogel. Whereas, the lack of physical interaction leads to poor substrate-to-nanomaterials adhesion [2,30]. Building upon this foundation, the integration of transition metal oxides onto cotton fabric for enhanced photocatalytic performance has gained prominence. Although some studies have explored the immobilization of transition metal oxides (TMOs) on fabric, the majority of these investigations have primarily focused on the utilization of titanium dioxide (TiO_2_). However, a noticeable research gap persists concerning the mechanistic understanding of the interaction between the fabric substrate and metal oxides. Additionally, there is a need for investigating the long-term stability and scalability of composite fabrics, particularly when exposed to commercial dyes. Moreover, it is crucial to extend our exploration beyond TiO_2_ and delve into the photocatalytic potential of other transition metal oxides and their heterostructures.

This study aims to bridge these gaps by addressing the limitations and challenges associated with conventional photocatalysts. Our focus lies in the exploration of immobilized heterostructure photocatalysts, with the goal of achieving enhanced performance, reduced agglomeration, and improved substrate-to-nanomaterial adhesion. Leveraging cellulosic textile waste fabric as the immobilizing substrate and employing bio-functionalization of CNT using organic acids, we propose a sustainable and eco-friendly approach to developing a crosslinked ZnO/fCNT/chitosan ternary heterostructure with superior photocatalytic capabilities. Through these advancements, our research contributes to the evolution of efficient and environmentally friendly wastewater treatment technologies, thereby paving the way for safer and more sustainable practices within the textile industry.

## 2. Results and Discussion

### 2.1. Analytical Assessments

**Figure 1:**
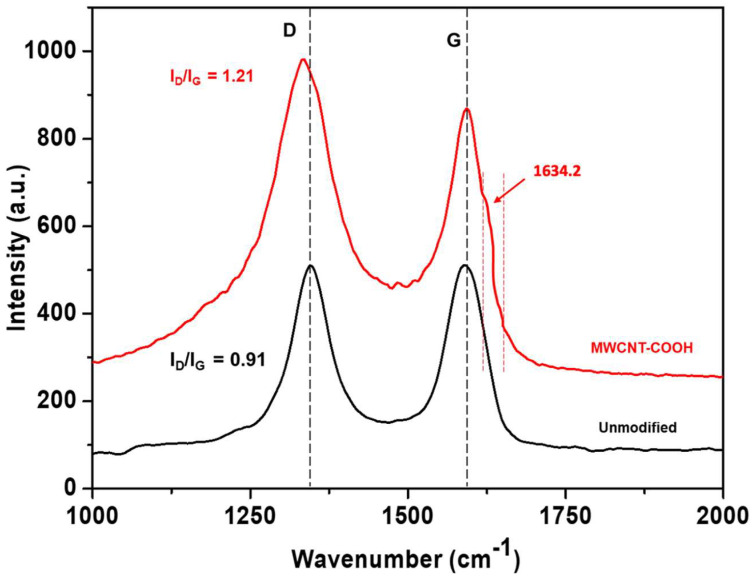
Raman spectra showing shifting of D and G bands of carbon nanotubes after acid treatment.

In this study, we prepared an immobilized ternary functional composite fabric by padding the fabric with functionalized carbon nanotubes (CNTs) incorporated with ZnO/chitosan dispersion. In the first step, the CNTs were bio-functionalized using organic acid (citric acid) to enhance their compatibility with the ZnO/chitosan system. This functionalization step is crucial for promoting proper dispersion and interaction between the CNTs in ZnO/Chitosan matrix and cotton substrate. To confirm the acid functionalization of multiwalled carbon nanotubes (MWCNT), Raman spectroscopy was utilized to investigate the surface modification effects. The obtained Raman spectra confirmed the successful acid functionalization of the CNTs and provided insights into the resulting changes in surface characteristics. The spectra exhibited distinct D and G bands, corresponding to nanotube vibrations roughly at 1341 cm^−1^ and 1582 cm^−1^, respectively (Figure 1. The shifting of the D and G bands in fCNT toward a lower wavenumber with observable change in bandwidths are indicative of an increase in the surface defect in CNT after acid treatment as reported in the literature (Figure 1; red line) [31]. The I_D_/I_G_ ratio is commonly used as the measure of the degree of disorder or defects present on the MWCNT surface. An increase in the I_D_/I_G_ ratio of fCNT (1.21) simply signifies the presence of surface defects and a lower degree of graphitization which is induced due to a shifting hybridization of C-C (sp_2_) to sp_3_. Further, the appearance of a shoulder signal near the G band (at 1634 cm^−1^) was also pronounced in the fCNT, which is possibly due to the developed -COOH functional groups by citric acid, which can readily graft onto the CNT structure [32].

**Figure 2:**
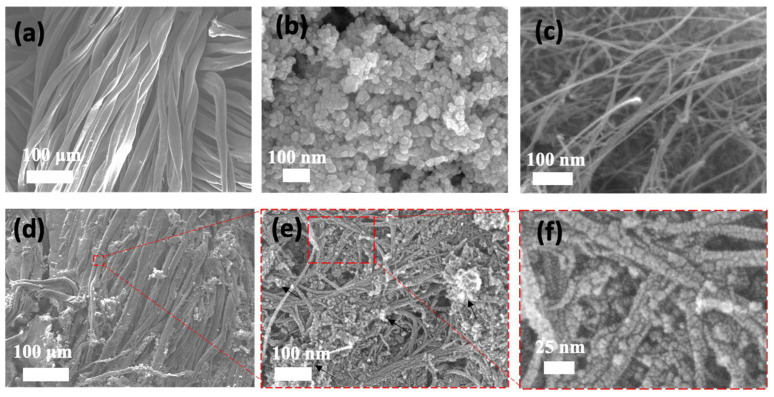
SEM Images showing surface morphology (**a**) Bare cotton fabric (**b**) ZnO (**c**) Carbon nanotubes (**d**–**f**) Composite fabric after treatment with ZnO/fCNT/Chitosan.

**Figure 3:**
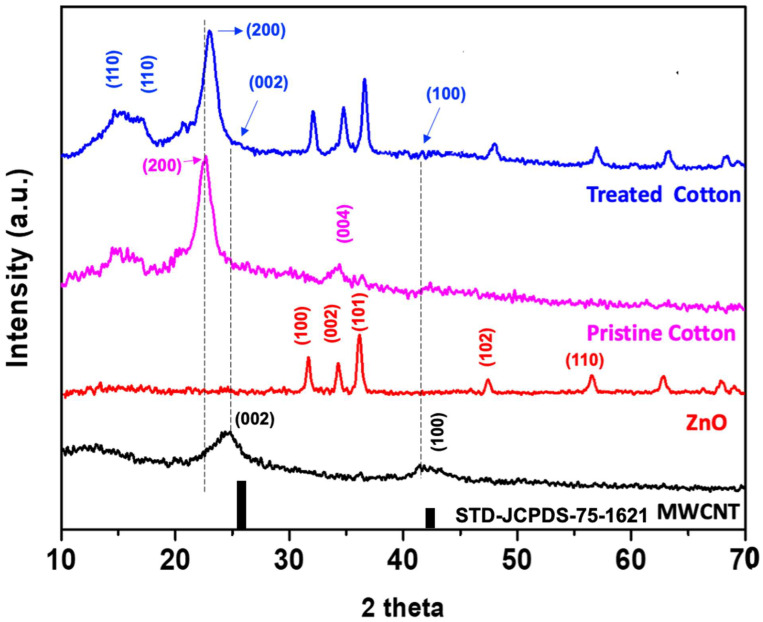
XRD spectra of functional carbon nanotube, ZnO nanoparticles, bare cotton fabric in comparison with as-prepared composite fabric and standard JCPDS of carbon nanotubes.

In the second step, a stable dispersion of ZnO/chitosan was prepared. Chitosan, a biocompatible and environmentally friendly polymer, was used as a matrix material to provide stability and adhesion properties with the composite fabric along with facilitating dye absorption. The functionalized CNTs were then added to the ZnO/chitosan dispersion, forming a ternary ZnO/CNT/chitosan dispersion. The prepared ZnO/CNT/chitosan dispersion was then applied to cotton fabric using a commercial pad dry cure method to create the immobilized functional ternary composite fabric [33]. Once prepared, scanning electron microscopy (SEM) analysis was performed to investigate the surface morphology and coating uniformity of the fabric samples. SEM images of the bare cotton fabric revealed a deconvoluted and smooth surface, typical of untreated cotton fibers (Figure 2a). Upon coating the fabric with the ZnO/CNT/chitosan dispersion, SEM images clearly revealed the presence of ZnO and CNT coatings. The coated fabric exhibited a textured surface, indicative of the successful immobilization of the ZnO and CNT composites onto the cotton fibers (Figure 2d–f). This visual confirmation suggests that the functionalized CNTs were effectively incorporated into the ZnO/chitosan matrix and subsequently adhered to the cotton fabric surface. The reference images of bare ZnO and CNT were also presented for review along with treated fabric (Figure 2b,c). To further validate the incorporation of ZnO and functionalized carbon nanotubes (fCNT) in the composite fabric, X-ray diffraction (XRD) analysis was performed. Figure 3 displays the XRD curves of the bare cotton fabric and the as-prepared composite fabric, along with reference spectra of fCNT and ZnO.

In the XRD spectrum of the bare cotton fabric, distinct diffraction peaks at 14.74°, 16.42°, and 22.7° were correspond to the (1–10), (110), and (200) planes of Cellulose-I, respectively [5]. The presence of these peaks confirms the crystalline nature of cellulose in the cotton fabric (Figure 3). Typically, due to its crystalline nature, MWCNT present the strong diffraction peaks nearly at ~25.7° and ~43.2° as reported in the literature (JCPDS card number 75–1621) [34]. The XRD spectrum of the functionalized carbon nanotubes (fCNT) exhibits broad diffraction peaks at 25.1° and 41.81°, corresponding to the (002) and (100) planes, respectively [32]. These broad and shifted peaks are attributed to defect sites generated in the carbon nanotubes after acid functionalization. The shifting of the (200) peak of Cellulose-I in composite fabric was observed due to the purlieu vicinity of ZnO and fCNT that reveals the structural interaction between ZnO, fCNT and cellulose (Figure 3) [10]. It can be seen that the composite fabric shows all the characteristic peaks of ZnO, confirming the presence of ZnO in the composite material. Additionally, the two peaks of functionalized carbon nanotubes (fCNT) were also observed in the composite fabric, although these peaks exhibited lower intensities when compared with the cellulose peaks. These weak graphitization carbon peaks in the composite fabric were attributed to the smaller amount of fCNT incorporated into the composite fabric compared to cellulose. The presence of ZnO and functionalized carbon nanotubes (fCNT) peaks in the composite fabric XRD spectrum confirms the successful integration of these materials into the fabric, validating the successful immobilization of ZnO and functionalized carbon nanotubes onto the cotton fabric.

**Figure 4:**
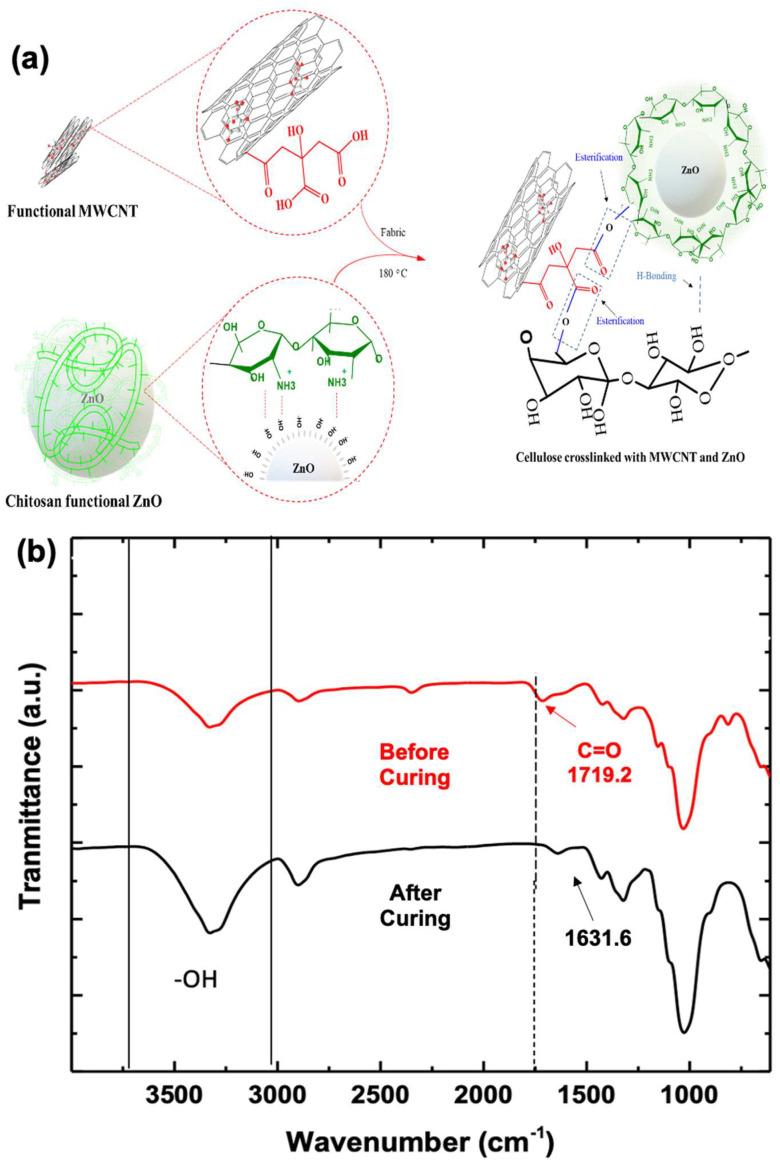
(**a**) Proposed reaction mechanism showing the bilateral esterification of fCNT with ZnO and cotton fabric at curing; (**b**) FTIR of the composite fabric before curing (black spectra) and after curing (red spectra) showing carbonyl peak.

To understand the immobilization of ZnO and functionalized carbon nanotubes (fCNT) onto the fabric surface, the development of heterojunctions was elucidated in the proposed reaction mechanisms. Raman spectroscopy revealed distinct shifts in the D and G bands of the CNTs, indicative of successful functionalization with citric acid. The -COOH groups introduced through the citric acid provided anchoring sites through bilateral esterification with the -OH group of chitosan, and cellulose consequently facilitated their subsequent immobilization onto the CNTs [32]. The citric-acid-functionalized CNTs acted as bridging agents between the ZnO nanoparticles and the fabric surface, enabling strong adhesion and anchoring of ZnO onto the fabric as a result of esterification and intermolecular interaction as proposed in the reaction scheme (Figure 4a). Further, in the Fourier transform infrared (FTIR) spectrum of the composite fabric, there was no carbonyl (C=O) peak before curing. The wide peak centered at 3320 cm^−1^ was attributed to -OH stretching, whereas short peak at 1631.6 cm^−1^ was indicative of absorbed water and unbonded carbonyl stretching (Figure 4b; black line) [35]. On curing, citric acid forms anhydrous particles and develops esterification with neighboring -OH of chitosan and cotton with the appearance of distinct carbonyl peaks at 1719.2 cm^−1^ [5]. The flatting of the -OH hump in between 3000 cm^−1^ and 3500 cm^−1^ further confirms the utilization of hydroxyl groups in esterification (Figure 4b; red spectra) [36]. The ester linkages play a crucial role in enhancing the adhesion and compatibility of the functionalized ZnO and carbon nanotubes with the fabric matrix, leading to improved stability and immobilization, consequently preventing agglomeration.

### 2.2. Photocatalytic Studies

**Figure 5:**
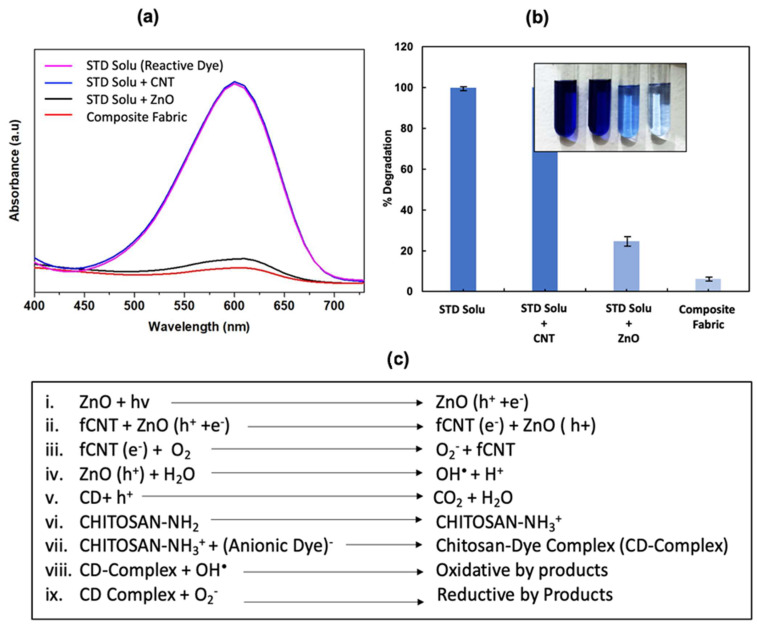
Photocatalytic studies of the composite fabric. (**a**) Degradation spectra of RB-4 after 4 h of irradiation; (**b**) Percentage degradation comparison of hybrid composite fabric in comparison with fCNT and ZnO; (**c**) Photocatalytic redox reactions presenting photodegradation of dye taking place at composite fabric surface.

The proposed photocatalytic mechanism of heterostructure-ZnO/fCNT/Chitosan- composite fabric was studied in comparison to bare ZnO and fCNT depicted in Figure 5.

In this study, we aimed to assess the photocatalytic performance of a composite fabric containing immobilized ZnO and functionalized carbon nanotubes (fCNT) in degrading the commercially used reactive blue 4 (RB-4) colorant. RB-4 is a highly resistant to degradation, posing significant environmental hazards [37]. To evaluate the photocatalytic activity, we prepared a 50 ppm RB-4 solution (50 mL) and placed a 1 × 1 inch composite fabric swatch in the solution. After achieving adsorption–desorption equilibrium in the dark for 30 min, the setup was exposed to direct sunlight for 4 h (Figure S1). The absorption spectra of the dye solution were then recorded using a UV–Vis spectrophotometer in the wavelength range of 350 to 750 nm. Figure 5a demonstrates that the addition of fCNTs alone in the solution exhibited no significant photocatalytic activity. The strong electrical conductivity of fCNTs hindered the separation of electron–hole pairs, thereby limiting photocatalytic performance [38,39]. In contrast, with the introduction of bare ZnO powder as a photocatalyst, electron–hole pairs are generated upon irradiation. The holes (h^+^) thus produced primarily engage in reactions with the adsorbed water (H_2_O) [40]. However, it is essential to recognize that these holes can also directly interact with the dye molecules, albeit predominantly preferring interaction with adsorbed water [41]. Moreover, the electrons (e^−^) participate in reactions with oxygen (O_2_), resulting in the formation of highly active hydroxyl radicals (^•^OH) and superoxide radical ions (O_2_^•−^) [25,42]. These radicals efficiently degraded the absorbed organic pollutant (RB-4). However, the photocatalytic activity of bare ZnO was hampered by rapid electron–hole recombination and nanoparticle agglomeration over time. The maximum degradation of 76.8% was recorded when ZnO powder (0.36 mg; 2% of the weight of fabric) was used alone as a photocatalyst (Figure 5b). In contrast to ZnO powder, the immobilized ZnO/fCNT/chitosan-coated composite fabric exhibited remarkable photocatalytic performance, achieving a color degradation of 93.54% which seems to be consistent in cycle utilization (Figure S2).

**Figure 6:**
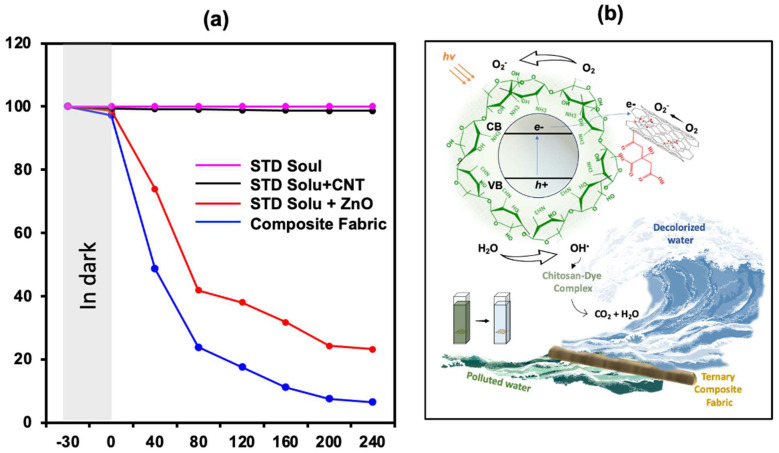
(**a**) Photodegrading rate (C_t_/C_o_) of 50 ppm solution of commercial reactive RB-4 vs. time; (**b**) Catalytic mechanism for photodegradation of RB-4 dye.

The degradation rate (C_t_/C_o_) verse time curve for immobilized fabric is presented in Figure 6. The improvement in color degradation was attributed to the immobilized nanocatalyst, which offers a large surface area for effective interaction with the pollutant [2]. Additionally, their strong adhesion with the substrate prevents particle agglomeration. These factors enhance catalytic efficiency and stability, making the immobilized nanocatalyst a promising approach for photocatalytic degrading. Chemically, upon irradiation, semiconductor materials (ZnO) provide the electrons to the system. In the ZnO/fCNT/chitosan junction, these electrons are radially accepted by fCNT, creating an effective pathway to suppress the electron–hole recombination. The adsorbed oxygen molecules on the nanotubes undergo a reaction with the electrons, leading to the formation of highly reactive superoxide radical ions (O_2_^•−^). These radical ions play a crucial role in the oxidation of the target compound (RB-4). Simultaneously, the holes (h^+^) generated during this process oxidize the hydroxyl groups, producing hydroxyl radicals (•OH) that also facilitate the decomposition of the target colorant [19]. Concomitantly. The interaction between RB-4 and chitosan merits attention. Owing to its anionic nature, RB-4 promptly interacts with chitosan through the potent electrostatic attraction between the negatively charged ionic dye and the positively charged amino groups of chitosan. This interaction leads to a substantial enhancement in dye absorption. As it is in the close vicinity of the ZnO/fCNT heterojunction, the absorbed dye (RB-4) is radially taken off by a photocatalyst and promptly reduced by hydroxyl radicals (•OH), as described in reaction equations (Figure 5c) [5]. Considering the dual mechanisms at play, it is observed that the initial adsorption of pollutants onto the photocatalyst surface is followed by their degradation through the generation of electron–hole pairs. The presence of chitosan amplifies the dye absorption process due to the pronounced ionic attraction between the dye and chitosan, resulting in enhanced degradation. The reduction in the band gap of ZnO in the presence of chitosan also contributes significantly by enhancing its ability to absorb visible light, thereby augmenting its tendency for photocatalytic degradation (Figure S3).

## 3. Material and Method

### 3.1. Materials

ZnO nanopowder (99.9%), Multiwall carbon nanotube (MWCNT) and C.I Reactive Blue 4 were purchased from Sigma Aldrich, Seoul, Republic of Korea. Citric acid anhydrous (99.5%) and chitosan were sourced from Dae-Jung Chemicals, Siheung-si, Republic of Korea; 100% cotton fabric weighing 180 g per m^2^ was kindly donated by Gohar Textile Mills, Faisalabad Pakistan.

### 3.2. Development of Immobilized Ternary Fabric Composite

**Figure 7:**
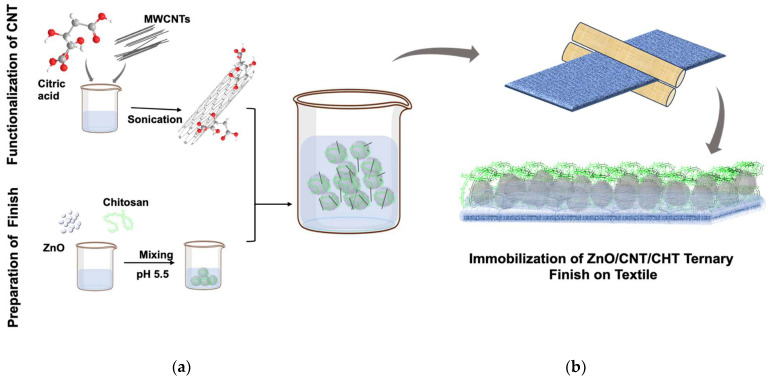
Schematic of development presenting synthesis of ternary composite fabric; (**a**) Synthesis of immobilization finish for textile application; (**b**) Application on fabric using padder.

For the development of ZnO/CNT/CHT ternary coated immobilized textile, first, CNT was functionalized using the method reported in the literature with slight modifications [32]. A total of 0.1 g of CNT were dispersed in a citric acid solution (10 g/100 mL) and sonicated for 6 h (at 1000 W and 20 kHz/kwatts @ 60 °C). After sonication time, the functional carbon nanotubes (fCNT) were thoroughly washed using double distilled water and centrifuged. In a separate beaker, 1 g of chitosan and 2.4 mg of ZnO nano-powder (per 1 cm^2^ of fabric) were dispersed in 100 mL of water. The pH of solution was set to be 5.5 (using acetic acid) while keeping the set up stirring at 400 revolutions per minute (RPM) for 30 min. After 30 min, 0.24 mg (1% of ZnO) of functional carbon nanotubes (fCNT) ware added in the beaker and the set was kept stirring for additional 30 min. Once homogenized, the fabric swatch (scoured and bleached) was padded using a padder with as-prepared ternary finishing solution at a wet pickup of 80%, followed by drying (at 100 °C for 5 min) and curing (at 180 °C for 2 min). The schematic of development is presented in Figure 7.

### 3.3. Characterizations

The immobilized ternary finish was applied on fabric using a Fabern-SP (China) laboratory padder.

Scanning electron microscopy (FE-SEM: JEOL, JSM-7600-F) was employed to investigate the surface morphology of the samples. FTIR spectra were analyzed using a Nicolet spectrophotometer (model: iD5-ATR). UV spectra were recorded using a Shimadzu UV-2600 spectrophotometer while Raman spectra were obtained using a Renishaw-2000 spectrometer. For RXD analysis, Bruker X-ray diffractometer D-8 Focus fitted with Copper Kα radiation with a λ value of 1.5418 Å was used (current = 40 mA; Voltage = 40 KV) in a scale range of 10°–70°.

## 4. Conclusions

The growing concerns surrounding environmental pollution and the urgent need for sustainable wastewater treatment have driven extensive research into advanced photocatalytic materials. Among these, immobilized photocatalysts have emerged as a promising solution to address persistent organic pollutants in industrial wastewater. This study explored the potential of a composite fabric incorporating immobilized ZnO and functionalized carbon nanotubes (fCNT) for the efficient degradation of the commercially used reactive blue 4 (RB-4) colorant. The results demonstrated a significant enhancement in photocatalytic performance with the inclusion of ZnO and fCNTs in the fabric along with chitosan immobilization. Raman spectroscopy provided compelling evidence of successful functionalization, evident from distinct shifts in the D and G bands of the CNTs due to the introduction of -COOH groups through citric acid treatment. FTIR spectroscopy further confirmed the functionalization process, revealing the appearance of carbonyl peaks at 1719.2 cm^−1^ in the composite fabric, in line with the proposed mechanism involving bilateral esterification of -COOH groups on the CNTs with -OH groups of chitosan and cellulose during the curing process. The immobilized nanocatalyst demonstrated remarkable photocatalytic performance, achieving an impressive 93.54% color degradation attributed to the suppression of electron–hole recombination and prevention of agglomeration through esterification. Conclusively, the ternary immobilized (ZnO/fCNT/chitosan) composite fabric presents a highly efficient and promising approach for the photocatalytic degradation of commercial dye and other hazardous pollutants in textile coloration processes. Future optimization of critical parameters, such as pH, temperature, and padding concentration, holds great potential for the development of environmentally friendly and sustainable wastewater treatment technologies in the textile industry, thereby contributing to a safer and more eco-friendly approach to address water pollution challenges.

## Data Availability

Data will be made available if demand.

## Supplementary Materials

The following supporting information can be downloaded at: https://www.mdpi.com/article/10.3390/molecules28186461/s1, Figure S1. Adsorption spectra of understudy sample including pristine cotton in dark. Figure S2. Cyclic reusability and stability and photocatalytic performance of composite fabric in consecutive cycles. Figure S3. Energy Band gap of ZnO before and after Chitosan Treatment (a) Bare ZnO (b) Chitosan treated ZnO, (c) Chitosan treated ZnO@MWCNT (Inset images present corresponding UV-Vis absorption spectra) [43].

## Sample Availability

Samples of the compounds are not available from the authors.
