# Supplementary material for: Harnessing Bio-Immobilized ZnO/CNT/Chitosan Ternary Composite Fabric for Enhanced Photodegradation of a Commercial Reactive Dye
Source: Molecules. 2023 Sep 6;28(18):6461. doi: 10.3390/molecules28186461 (PMC10536000; doi:10.3390/molecules28186461)
Supplement: Supplementary file 1 [file molecules-28-06461-s001.zip › molecules-2560296-supplementary.pdf]

Harnessing Bio-Immobilized ZnO/CNT/Chitosan Ternary Composite Fabric for Enhanced Photodegradation of a Commercial Reactive Dye

Usama Bin Humayoun [a†], Fazal Mehmood [a†], Yasir Hussain [b†], Aamir Rasheed [c], Ghulam Dastgeer [d],Asad Anwar [a], Nasir Sarwar [a]*, Dae Ho Yoon [e,f] *

[a] Department of Textile Engineering, University of Engineering & Technology, Lahore (Faisalabad Campus), 38000, Pakistan.

[b] Department of Materials Science and Engineering, Chungnam National University, South Korea.

[c] School of Materials Science and Engineering, Anhui University, China.

[d] School of Physics, Sejong University, South Korea

[e] School of Advanced Materials Science and Engineering, Sungkyunkwan University, Suwon 16419, Republic of Korea.

[f] SKKU Advanced Institute of Nanotechnology (SAINT) Sungkyunkwan University, Suwon 16419, Republic of Korea.

† These authors contributed equally to this work

* Corresponding authors. Tel.: +82-31-290-7388; fax: +82-31-290-7371. E-mail address:
Nasir.sarwar@uet.edu.pk
dhyoon@skku.edu

ZnO weight calculation for 2% of the weight of fabric

Weight of fabric $1m^2$	= 180 g
Weight of (1x1)" fabric swatch	= 180 / 1550 g
	= 0.12 g
Weight of ZnO powder used in reference experiment	= 2% of the weight of fabric
	= 2 % of 0.12
	= 0.0024 g
	= 2.4 mg

The photo-stability of the immobilized composite fabric was evaluated as the function of reusability. The immobilized fabric was subjected to four consecutive cycles of utilization. Following each trial, the utilized fabric segment underwent a comprehensive washing under tap water prior utilization in subsequent experiments. The immobilized fabric exhibited an effective stability and reusability across these successive trials. It In fourth trial, the fabric maintained an effective photodegradation efficiency of 89.2%. Furthermore, a nearly comparable level of photocatalytic performance was demonstrated throughout these trials, with only a marginal 4.6 % reduction in efficiency. This observation strongly validates the enduring reusability and stability of the immobilized ternary fabric composite.

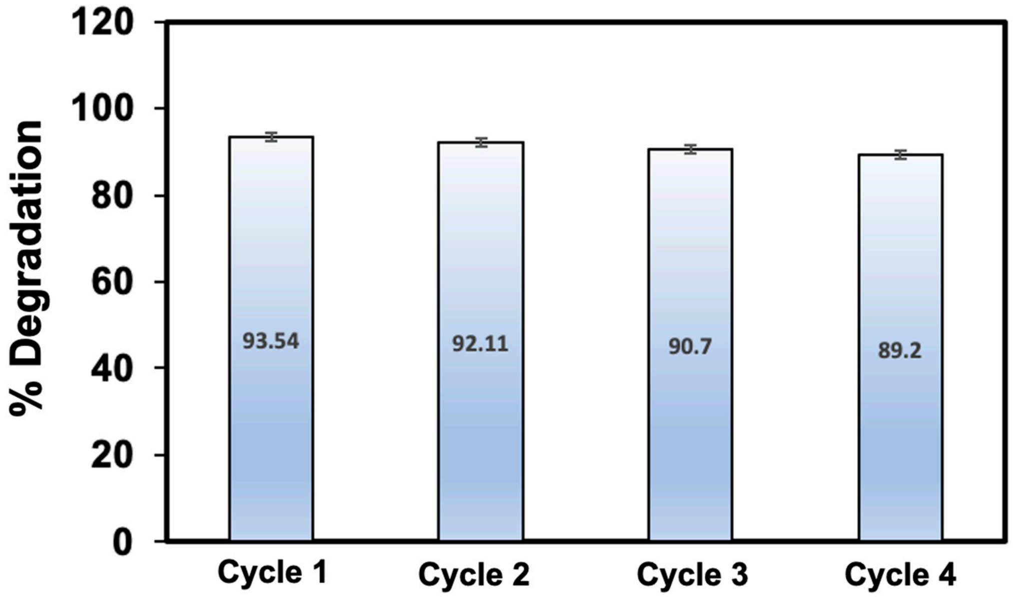

Figure S1. Cyclic reusability and stability and photocatalytic performance of composite fabric in consecutive cycles.

The calculated band gaps and absorption spectra (inset) have been presented in figure S2. The band gap value of the pristine ZnO was found to be 3.3 eV which absorb high-energy light. However, upon chitosan interaction the band gap values decreased to 2.3 eV. A smaller band gap energy can result in increased photon absorption, leading to the generation of higher production of reactive oxidative species consequently enhanced photocatalytic activity [1].

[1] Gao, X., Li, L., An, M., Zheng, T. & Ma, F. (2022) ZnO QDs and three-dimensional ordered macroporous structure synergistically enhance the photocatalytic degradation and hydrogen evolution performance of WO3/TiO2 composites. Journal of Physics and Chemistry of Solids, 165, 110655.

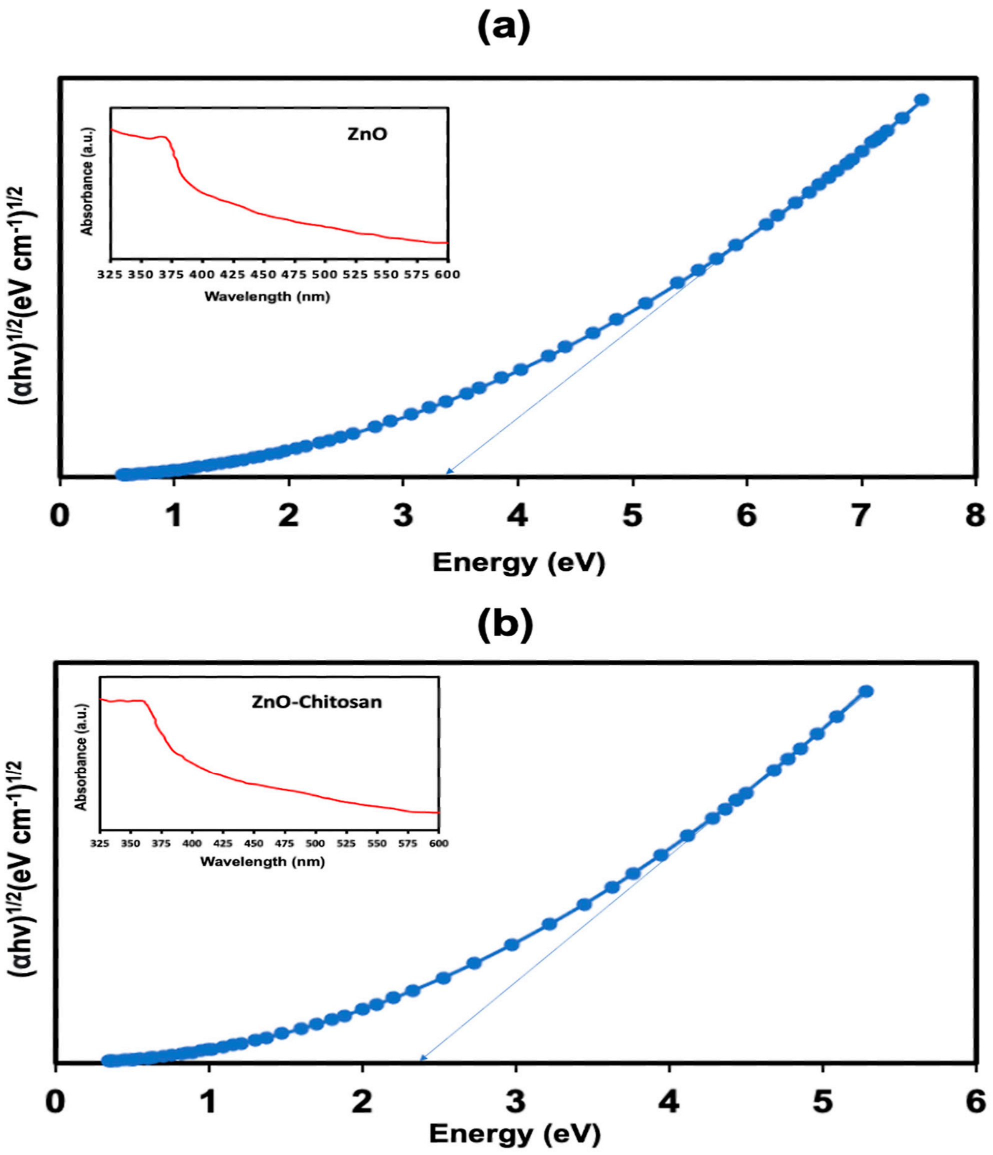

Figure S2. Energy Band gap of ZnO before and after Chitosan Treatment (a) Bare ZnO (b) Chitosan treated ZnO, Inset image present corresponding UV-Vis absorption spectra.
